# Predictors of Recurrence After Surgery in Patients with Stage I Non-Small Cell Lung Cancer

**DOI:** 10.3390/cancers18071152

**Published:** 2026-04-03

**Authors:** Emanuele Voulaz, Debora Brascia, Veronica Giudici, Stefano Margaritora, Marco Lucchi, Vittorio Aprile, Marco Chiappetta, Alexandro Patirelis, Vincenzo Ambrogi, Giuseppe Marulli

**Affiliations:** 1 IRCCS Humanitas Research Hospital, 20089 Rozzano, Italy; 2 Humanitas University, 20072 Pieve Emanuele, Italy; 3 Fondazione Policlinico Universitario “A. Gemelli” IRCCS, 00168 Rome, Italy; 4 Azienda Ospedaliero-Universitaria Pisana, 56124 Pisa, Italy; 5 Thoracic Surgery Unit, Magna Graecia University, 88100 Catanzaro, Italy; 6 Tor Vergata University Hospital, 00133 Rome, Italy

**Keywords:** lung cancer surgery, robotic, VATS, thoracotomy

## Abstract

**Background:** Surgery represents the gold standard treatment for patients with stage I non-small cell lung cancer (NSCLC); however, up to 30% of those may experience recurrence. This study aims to identify prognostic factors for both early and late recurrence in this subset. **Methods:** We retrospectively analyzed the data of patients with stage IA-B NSCLC undergoing lung resection from 2013 to 2021 in four major lung cancer centers. Inclusion criteria were intentionally curative resections via either open or mini-invasive approaches plus lymph node dissection and a minimum follow-up of 36 months. Analyzed prognosticators included age, gender, smoking status, comorbidities, radiological appearance, surgical approach, intraoperative complications, pT stage and histologic subtypes. The overall and disease-free survivals and uni- and multivariable Cox regression for recurrence prediction were analyzed. **Results:** We collected data from 1132 consecutive patients (mean age 68.5 ± 8.8 years, 55.5% males and 20.1% smokers). After a mean follow-up of 57 ± 37 months, 908 (80.2%), patients were still disease-free, while the remaining 224 (19.8%) presented local (*n* = 86) or distant (*n* = 138) recurrences; 72 (32.1%) patients experienced reoccurrence within 12 months. The disease-free survival rate was significantly higher in the pT1a stage and in lepidic adenocarcinoma. The multivariable analysis and Cox regression showed that pT>1a (*p* = 0.001) and non-lepidic subtypes of adenocarcinoma (*p* < 0.001) were the best predictors of recurrence. **Conclusions:** Approximately one fifth of patients undergoing radical surgery for stage I NSCLC experienced recurrence within five years. Significant predictors of recurrence were a pT status greater than 1a and non-lepidic subtypes of adenocarcinoma.

## Simple Summary

Surgery represents the gold standard treatment for patients with stage I non-small cell lung cancer (NSCLC); however, up to 30% of those may experience recurrence. This study aims to identify prognostic factors for both early and late recurrence in this subset. We retrospectively analyzed data of 1132 patients with stage IA-B NSCLC undergoing lung resection from 2013 to 2021. All the patients were treated with radical surgery, and approximately 20% of cases recurred within five years. Significant predictors of recurrence were an open approach, pT status greater than 1a and non-lepidic subtypes of adenocarcinoma.

## 1. Introduction

Lung cancer remains one of the most common malignancies worldwide, with approximately 1.8 million new diagnoses, accounting for about 13% of all cancer cases diagnosed [1]. Surgical resection represents the cornerstone of the treatment of locoregionally confined non-small cell lung cancer (NSCLC; clinical stages I to III) [2]. Despite curative intentions, approximately 40–55% of lung cancer patients experience recurrence, depending on the stage of the disease [3,4,5,6]. Several studies have documented recurrence in patients who underwent surgery for stage I NSCLC. Martini et al. [7] investigated 598 patients diagnosed with stage I NSCLC and found a recurrence rate of 27%, with 60% of these recurrences occurring within two years post-surgery. Maeda et al. [8] examined 483 patients who remained recurrence-free for five years following surgery, discovering that 4.8% experienced a delayed recurrence more than 5 years following their procedure. An improved risk stratification for these patients could justify the intensification of adjuvant therapies for a subgroup of early-stage lung cancer patients deemed to be at a high risk for recurrence [9]. Nevertheless, none of these studies have identified the specific factors associated with early recurrence or identified reliable prognostic indicators following recurrence.

This study aims to identify the prognostic factors associated with early and late recurrence in patients who have undergone radical surgery for stage I NSCLC.

## 2. Materials and Methods

We reviewed the medical records of patients from four major lung cancer centers who underwent surgical treatment for clinical stage I NSCLC from 2013 to 2021.

Complete resection was defined as a radical procedure without any macroscopic residual lesions and with a microscopically clear margin, associated with a systematic lymph node dissection including a minimum of six nodes/stations, three of which had to be mediastinal, always including the sub-carinal station. Wedge resections with an adequate margin greater than 10 mm were also considered radical in those patients with limited residual pulmonary function and for lesions less than 15 mm in diameter. Only patients with confirmed pN0 status were eligible for analysis to ensure accuracy of the final pathological stage. Patients with multiple lung cancers (either synchronous or metachronous) and those who underwent incomplete resection were excluded from this analysis.

All patients had a histological diagnosis of NSCLC before or during surgery. Surgical approaches included open thoracotomy, video-assisted thoracic surgery (VATS), and robotic-assisted thoracic surgery (RATS). All resected specimens underwent pathological examination. The histological classification included a subdivision of adenocarcinoma histotypes, and the pathologic T factor was reclassified according to the eighth edition of the Tumors, Node and Metastasis (TNM) classification system described by the International Association for the Study of Lung Cancer (IASLC) [10,11].

Collected variables included: age, gender, body mass index (BMI), comorbidities (yes/no), smoking history (ever or never), imaging appearance of the tumor on computed tomography (CT) scans (solid, ground-glass opacity, mixed), resection volume (wedge, segmentectomy, lobectomy or bilobectomy), surgical approach (open, VATS or RATS), and pT status. Early recurrence was defined as any recurrence occurring within 12 months after surgery. Local recurrence was defined as tumor relapse in the same chest cavity (including mediastinal structures and chest wall), while distant recurrence referred to tumor appearance in the other organs.

Data collected and reported in this study were retrieved from patient’s medical records and pathological and surgical registries. Follow-up data were obtained through outpatient visits, imaging studies (total body CT scan or FDG-PET scan) and phone interviews. To account for potential inter-institutional variability and temporal changes in surgical practice, additional sensitivity analyses were performed including center of treatment and calendar year of surgery as covariates in multivariable models. The data collection for this retrospective analysis was approved by the institutional review board (IRB) of Humanitas Research Hospital, and individual patient consent was obtained to authorize the use of their clinical data for research purposes and retrospective analysis in an anonymous form. The STROBE reporting recommendations were adopted in the reporting of this study [12].

### Statistical Analysis

Descriptive statistics were used to summarize the demographic, clinical, surgical, and pathological characteristics of the cohort. Continuous variables were reported as mean ± standard deviation (SD) and compared using the Wilcoxon rank-sum test. Categorical variables were presented as counts and percentages, and differences between groups were assessed using the chi-square test or Fisher’s exact test, as appropriate. Firstly, the association of major variables with the time (early or late) and site (local or distant) of the recurrence was investigated. Then, disease-free survival (DFS) and overall survival (OS) were estimated using the Kaplan–Meier method. DFS was defined as the time from surgery to the first recurrence (local, distant, or both) or last follow-up. DFS in the presence/absence of different factors was compared with the log-rank test. The Cox proportional hazards model was used to determine independent predictors of recurrence. Univariate analyses were performed to identify variables potentially associated with recurrence. Variables with a *p* < 0.10 in the univariate analysis were included in the multivariate analysis. Hazard ratios (HRs) with 95% confidence intervals (CIs) were calculated. BMI was treated as a purely descriptive variable for the available subset and was not included in any further statistical testing.

All tests were two-sided, and a *p*-value < 0.05 was considered statistically significant. Statistical analyses were conducted using STATA v17.0 (StataCorp, College Station, TX, USA).

## 3. Results

We retrieved data from a total of 1132 patients; the main demographic, surgical and pathological features are summarized in Table 1.

A lobectomy was performed in 995 (88%) patients, an anatomic segmentectomy was performed in 56 (4%) and a wedge procedure was performed in 81 (7%). The majority of the operations (57.1%) were carried out through a minimally invasive approach: 552 VATSs and 106 RATSs, respectively. The open approach consisted of a standard lateral thoracotomy.

The histological classification revealed adenocarcinoma in 920 (81.3%) patients and squamous cell carcinoma in 141 (12.5%) patients. According to eighth edition of the TNM classification, 212 (18.7%) patients had stage pT1a, 402 (35.5%) had stage pT1b, 223 (19.7%) had stage pT1c and 295 (26.1%) had stage pT2a.

All the patients were followed over time with a mean follow-up of 57 ± 37 months.

### 3.1. Recurrences

Recurrence were observed in 224 patients (19.8%), of which 86 were local, 106 were distant-only and 32 involved both local and distant sites. Patients who experienced both local and distant recurrences simultaneously were classified and counted only within the ‘distant recurrence’ group, as this reflects the most clinically significant event.

In 72 (32.1%) patients, the recurrence occurred within one year after surgery. Patients who developed early recurrence were evaluated through a multidisciplinary approach. In cases where the recurrence was isolated, a local treatment with radical intent was considered.

Several factors were associated with early recurrence (Table 2). However, a multivariable logistic regression analysis was performed to identify independent predictors of early recurrence (Table 3). After adjustments for age, sex, the pT status, histology, and the year of surgery, the surgical approach was not independently associated with early recurrence. In contrast, later pT stages and squamous histology were significantly associated with an increased risk of early recurrence, while RATS showed a statistically significant association with early recurrence. This finding should be interpreted cautiously due to the small sample size and large confidence intervals.

Regarding the site of recurrence, distant relapses were more frequent in patients undergoing lobar resection (*p* = 0.0001) or receiving an open approach (*p* = 0.007) or with a pT2a tumor (*p* = 0.008) (Table 4).

### 3.2. Survival Analysis

**Figure 1:**
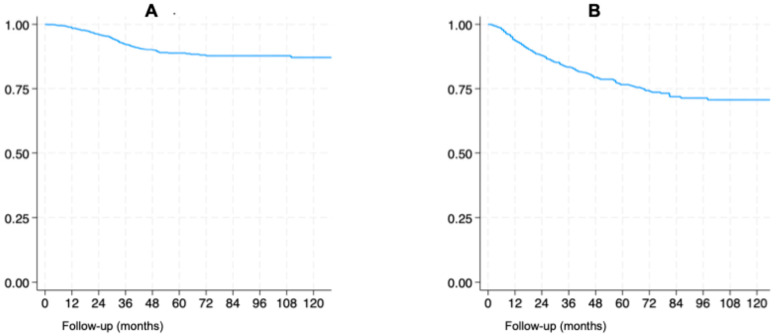
Overall survival (**A**) and disease-free survival (**B**).

Overall survival rates at 12, 36, and 60 months were 98.4%, 92.1% and 89.3% (Figure 1A), respectively. Disease-free survival rates for the same intervals were 93.5%, 83.3% and 76.5%, respectively (Figure 1B).

**Figure 2:**
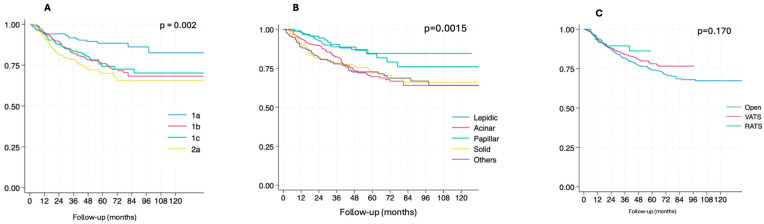
Disease-free survival by pathological T Status (8th TNM) (**A**), adenocarcinoma subtypes’ histology (**B**) and surgical approach (**C**). *p*-values were calculated using the global log-rank test.

The disease-free survival rate was significantly higher for pT1a (*p* = 0.002, Figure 2A) and for the lepidic/papillar adenocarcinoma histology (*p* = 0.0015, Figure 2B). VATS/RATS resections had better outcomes compared to the open approach, although these were not significant (*p* = 0.170, Figure 2C).

### 3.3. Cox Regression

In the univariable analysis, patients who developed recurrence were more commonly operated on via an open approach (*p* < 0.001), with a pT stage above 1a (*p* < 0.001) and non-lepidic adenocarcinoma subtypes (*p* < 0.001) (Table 5).

The multivariable analysis showed that an open surgery (*p* = 0.032), pT stage > 1a (pT1b *p* = 0.036; pT1c *p* = 0.052; pT2a *p* = 0.003) and non-lepidic subtype among adenocarcinomas (acinar: *p* = 0.012; solid: *p* = 0.016, other: *p* = 0.008) were independent predictors of recurrence (Table 5). In the primary multivariable model, the surgical approach appeared to be associated with the recurrence risk. In this adjusted model, the association between the surgical approach and recurrence was attenuated and no longer statistically significant (VATS vs open: HR 1.37, 95% CI 0.94–2.01, *p* = 0.102; RATS vs open: HR 1.97, 95% CI 0.91–4.27, *p* = 0.087). The calendar year showed a modest association with the recurrence risk (HR 1.09 per year, 95% CI 1.01–1.18, *p* = 0.025). Full results are reported in Supplementary Table S1.

## 4. Discussion

Despite having radical surgery, patients with completely resected node-negative NSCLC remain at risk for recurrence. Many retrospective studies have analyzed the risk factors for relapse in stage I NSCLC following radical resection and have attempted to develop a risk prediction model based on clinical factors and biomarkers [13,14,15,16,17,18,19,20,21,22].

In our cohort of 1132 of patients, the 5-year recurrence rate was 19.8%, consistent with previous reports [23,24]. We found that the pathological stage, particularly stages greater than IA, significantly contributed to the risk of recurrence. This aligns with findings from Zhao et al. [25], who reported similar conclusions in their study of stage I lung adenocarcinoma. We also identified strong associations between other clinic-pathological features and recurrence, specifically adenocarcinoma subtypes. Our analysis also showed that the non-lepidic subtype of adenocarcinoma was linked to a higher risk of recurrence within five years, and the multivariate analysis confirmed this finding. A recent validation study of the IASLC histologic grading system also supported these findings [16]. Woo et al. [26] demonstrated that grade 3 tumors and non-lepidic subtypes were associated with worse recurrence-free survival and overall survival [20,26].

Our analysis suggested that patients undergoing open thoracotomy may have a higher recurrence risk. However, after additional adjustments for the center and calendar year, this association is no longer significant, and the direction changes. This finding is not widely corroborated in the current literature [27]. At least several studies have suggested that the open surgery is the safer operation, and the minimally invasive approach is not inferior in terms of overall survival or the risk of recurrence in early-stage lung cancer [28,29,30]. The apparent association between the surgical approach and recurrence should therefore be interpreted cautiously. In retrospective surgical series, an open thoracotomy is often selected for patients with more complex or radiologically suspicious tumors, which may inherently carry a higher risk of recurrence. In addition, given the retrospective design and the long inclusion period (2013–2021), open surgery was more frequently performed in the earlier years of the study period, when minimally invasive approaches were less widely adopted and surgeon expertise with VATS or robotic techniques was still evolving across centers.

Our findings indicate that the association between the surgical approach and outcomes is model-dependent and loses statistical significance after adjustments for the center and calendar year, suggesting that institutional and temporal factors may partly explain the observed differences.

As far as the resection volume, we found that sublobar resection was not associated with an increased rate of local or distant recurrence. Similarly, several studies suggest that a sublobar resection may be equivalent to a lobectomy in a selected subset of patients, such as ground-glass opacities and well-differentiated tumors less than 2 cm [31,32,33]. On the other hand, Varlotto et al. reported an increase in local recurrence rates among patients who underwent a sublobar resection for moderately differentiated NSCLC tumors larger than 2 cm [34], while El-Sherif et al. found that patients with stage IB NSCLC had worse disease-free survival rates when treated with sublobar resection compared to the standard lobectomy [35]. The low percentage of sublobar resections (12.1%) in our series may suggest a strict selection of patients that justifies the results being comparable to lobar resection.

Based on the survival analysis presented in our study, the possibility of offering adjuvant therapy to selected patients should be considered, even when pathological staging indicates early-stage oncologic disease.

### Limitations

The main limitation of our study is its retrospective design, as selection bias can play a role that cannot be reliably controlled. Furthermore, due to the multicenter nature of this study, the choice of surgical approach or the extent of the pulmonary resection may be influenced by the center of origin; in particular, the variable availability of robotic and minimally invasive platforms across centers may have resulted in a heterogeneous distribution of cases and should therefore be considered a potential confounding factor. We developed our multivariable analysis by selecting variables that were reported in the literature and supported by our clinical experience as potential predictors of recurrence. However, given the sample size, we were limited to including only five variables in the multivariable model.

The histopathologic classification of lung tumors has undergone major changes over the past decade, and certain features (such as spread through airspaces, STAS) were not systematically recorded at the start of our study. Given the prognostic relevance of STAS [36], further research is warranted as its reporting becomes increasingly standardized.

## 5. Conclusions

In our analysis, the 5-year recurrence rate is comparable to that reported in the literature, and we demonstrated that the disease-free survival rate was significantly worse for patients at stages greater than pT1a and in those with non-lepidic adenocarcinoma.

## Figures and Tables

**Table 1:** Clinico-pathologic characteristics of all patients (*n* = 1132).

Variables	Value	%
Age at surgery (mean ± SD)	68 ± 8.8	
Gender (male)	629	55.5%
Body mass index (kg/m^2^) *	24.6 ± 5.4	
Comorbidity (none)	300	26.5%
Smoke		
Never	400	35.3%
Ex	495	43.7%
Current	227	20.1%
Unknown	10	0.9%
CT appearance		
Solid	758	67%
Ground-glass	164	14.5%
Mixed	147	13%
Unknown	63	5.5%
Surgical approach		
Open	474	41.9%
VATS	552	48.7%
RATS	106	9.4%
Resection volume		
Lobectomy/bilobectomy	995	87.9%
Segmentectomy	56	4.9%
Wedge	81	7.2%
pT status (8th TNM)		
1a	212	18.7%
1b	402	35.5%
1c	223	19.7%
2a	295	26.1%
Histology		
Adenocarcinoma	920	81.3%
Squamous carcinoma	141	12.5%
Other	71	6.2%
Subtype of adenocarcinoma		
Lepidic	285	25.2%
Acinar	264	23.3%
Papillar	95	8.4%
Solid	147	13%
Other	134	11.8%
NA	207	18.3%

* These data refer only to 761 patients. NA: missing data.

**Table 2:** Univariate analysis of demographic and clinico-pathologic features of patients with early and late recurrence.

	Univariate Analysis		
	Early Recurrence	Late Recurrence	*p*
N	72	152	
Age	73 (60–77)	70 (61–77)	0.015
Gender (male)	52 (72.2%)	89 (58.5%)	0.048
Surgical approach			0.005
Open	29 (40.3%)	89 (58.6%)	
VATS	35 (48.6%)	59 (38.8%)	
RATS	8 (11.1%)	4 (2.6%)	
Resection volume			0.469
Lobectomy/bilobectomy	59 (81.9%)	132 (86.9)	
Segmentectomy	3 (4.2%)	7 (4.6%)	
Wedge	10 (13.9%)	13 (8.5)	
pT status (8th TNM)			0.246
1a	11 (15.3%)	11 (7.2%)	
1b	23 (31.9%)	60 (39.5%)	
1c	13 (18.1%)	31 (20.4%)	
2a	25 (34.7%)	50 (32.9%)	
Histology			0.029
Adenocarcinoma	54 (75%)	132 (86.8%)	
Squamous carcinoma	13 (18.1%)	10 (6.6%)	
Other	5 (6.9%)	10 (6.6%)	
Subtype of adenocarcinoma			0.118
Lepidic	6 (8.3%)	25 (16.3%)	
Acinar	16 (22.2%)	50 (32.7%)	
Papillar	2 (2.8%)	12 (7.8%)	
Solid	15 (20.8%)	24 (16.3%)	
Other	15 (20.8%)	23 (15%)	
NA	18 (25.1%)	18 (11.9%)	

NA: missing data.

**Table 3:** Multivariable logistic regression for early recurrence (≤12 months).

Variable	Adjusted OR	95% CI	*p*-Value
**Surgical Approach**			
VATS vs Open	1.59	0.79–3.20	0.190
RATS vs Open	4.81	1.16–19.87	0.030
**pT Status**			
1b vs 1a	0.20	0.07–0.59	0.004
1c vs 1a	0.19	0.06–0.65	0.008
2a vs 1a	0.23	0.08–0.71	0.010
**Histology**			
Squamous vs Adeno	2.72	1.05–7.03	0.039
Other vs Adeno	1.32	0.41–4.32	0.642
Age	1.03	0.99–1.07	0.114
Female vs Male	0.57	0.30–1.10	0.094
Year	1.08	0.94–1.24	0.265

**Table 4:** Univariate analysis of demographic and clinico-pathologic features of patients with local and distant recurrence.

	Univariate Analysis		
	Local Recurrence	Distant * Recurrence	*p*
N	86	138	
Age	73 (60–77)	70 (61–77)	0.059
Gender (male)	57 (66.2%)	84 (60.8%)	0.425
Surgical approach			0.007
Open	53 (61.6%)	65 (47.1%)	
VATS	33 (38.4%)	61 (44.2%)	
RATS	0 (0%)	12 (8.7%)	
Resection volume			0.0001
Lobectomy/bilobectomy	63 (73.3%)	128 (92.7%)	
Segmentectomy	7 (8.1%)	3 (2.2%)	
Wedge	16 (18.6%)	7 (5.1%)	
pT status (8th TNM)			0.008
1a	14 (16.3%)	8 (5.8%)	
1b	35 (40.7%)	48 (34.8%)	
1c	18 (20.9%)	26 (18.8%)	
2a	19 (22.1%)	56 (40.6%)	
Histology			0.198
Adenocarcinoma	76 (88.4%)	110 (79.7%)	
Squamous carcinoma	7 (8.1%)	16 (11.6%)	
Other	3 (3.5%)	12 (8.7%)	
Subtype of adenocarcinoma			0.084
Lepidic	6 (7%)	25 (18.1%)	
Acinar	28 (32.5%)	38 (27.5%)	
Papillar	8 (9.3%)	6 (4.3%)	
Solid	16 (18.6%)	23 (16.7%)	
Other	18 (20.9%)	20 (14.5%)	
NA	10 (11.7%)	26 (18.9%)	

* Including patients who experienced both distant-only and local and distant recurrences simultaneously. NA: missing data.

**Table 5:** Univariable and multivariable analyses for recurrence in the overall cohort of patients (Cox proportional hazards model).

	Univariate Analysis			Multivariate Analysis	
	Recurrence	No Recurrence	*p*	HR (95%CI)	
N	224	908			
Age	69.52 ± 9.44	68.25 ± 8.76	0.057	1.01 (0.99–1.03)	0.184
Gender (male)	141 (62.9%)	488 (53.7%)	0.013	0.67 (0.49–0.91)	0.010
Body mass index (kg/m^2^) *	24.7 ± 3.6 (N = 124)	24.6 ± 3.1 (N = 637)	0.802		
Comorbidity	53 (34.7%)	247 (37.0%)	0.315		
Smoke			0.482		
Never	78 (35.3%)	322 (35.7%)			
Ex	104 (47.1%)	391 (43.4%)			
Current	39 (17.7%)	188 (20.9%)			
CT appearance			0.285		
Solid	166 (74.7%)	592 (69.9%)			
Ground-glass	32 (14.4%)	132 (15.6%)			
Mixed	24 (10.8%)	123 (14.5%)			
Surgical approach			<0.001		
Open	118 (52.7%)	356(39.0%)		1 (ref)	
VATS	94 (42.0%)	458 (50.4%)		0.71 (0.52–0.97)	0.032
RATS	12 (5.4%)	94 (10.3%)		0.57 (0.29–1.17)	0.169
Resection volume			0.127		
Lobectomy/bilobectomy	191	804			
Segmentectomy	23	58			
Wedge	10	46			
Intraoperative complications	63 (28.1%)	227 (25.1%)	0.508		
pT status (8th TNM)			<0.001		
1a	22(9.8%)	190 (20.9%)		1 (ref)	
1b	83 (37.0%)	319 (35.1%)		1.68 (1.03–2.72)	0.036
1c	44 (19.6%)	179 (19.7%)		1.70 (1.00–2.89)	0.052
2a	75 (33.5%)	220 (24.2%)		2.16 (1.31–3.55)	0.003
Histology			0.557		
Adenocarcinoma	186 (83.0%)	735 (80.9%)			
Squamous carcinoma	23 (10.3%)	117 (12.9%)			
other	15 (6.7%)	56 (6.2%)			
Subtype of adenocarcinoma			<0.001		
Lepidic	31 (13.8%)	254 (28.0%)		1 (ref)	
Acinar	66 (29.5%)	198 (21.8%)		1.76 (1.13–2.73)	0.012
Papillar	14 (6.2%)	81 (8.9%)		0.97 (0.51–1.85)	0.931
Solid	39 (17.4%)	108 (11.9%)		1.81 (1.12–2.93)	0.016
Not applicable	38 (17.0%)	96 (10.6%)		1.97 (1.19–3.25)	0.008

* These data refer only to 761 patients.

## Data Availability

The original contributions presented in this study are included in the article/Supplementary Materials. Further inquiries can be directed to the corresponding author.

## Supplementary Materials

The following supporting information can be downloaded at https://www.mdpi.com/article/10.3390/cancers18071152/s1, Table S1. Sensitivity multivariable Cox model for recurrence, additionally adjusted for center and calendar year of surgery.

## Abbreviations

The following abbreviations are used in this manuscript:

NSCLC	Non-Small Cell Lung Cancer
VATS	Video-Assisted Thoracic Surgery
RATS	Robotic-Assisted Thoracic Surgery
TNM	Tumors, Node and Metastasis
CT	Computed Tomography
FDG-PET	Fluorodeoxyglucose Positron Emission Tomography
DFS	Disease-Free Survival
OS	Overall Survival
STAS	Spread Through Airspaces
