# Supplementary material for: Predictors of Recurrence After Surgery in Patients with Stage I Non-Small Cell Lung Cancer
Source: Cancers (Basel). 2026 Apr 3;18(7):1152. doi: 10.3390/cancers18071152 (PMC13072170; doi:10.3390/cancers18071152)
Supplement: Supplementary file 1 [file cancers-18-01152-s001.zip › cancers-4194050-supplementary.pdf]

Supplementary Table S1. Sensitivity multivariable Cox model for recurrence, additionally adjusted for center and calendar year of surgery.

Variable	HR	95% CI	*p*-Value
Female vs Male	0.59	0.42–0.83	0.003
VATS vs Open	1.37	0.94–2.01	0.102
RATS vs Open	1.97	0.91–4.27	0.087
pT1b vs pT1a	0.60	0.35–1.03	0.066
pT1c vs pT1a	0.65	0.36–1.16	0.146
pT2a vs pT1a	0.70	0.40–1.23	0.220
Adeno Acinar vs Lepidic	0.95	0.57–1.58	0.832
Adeno Papillar vs Lepidic	0.78	0.40–1.54	0.478
Adeno Solid vs Lepidic	1.07	0.64–1.79	0.801
Adeno Ohers vs Lepidic	1.13	0.62–2.05	0.685
Age (per year)	1.01	0.99–1.02	0.374
Center 1 vs center 2 *	1.04	0.67–1.61	0.852
Center 3 vs center 2 *	0.81	0.10–6.32	0.837
Center 4 vs center 2 *	0.87	0.51–1.46	0.590
Year of surgery (per year)	1.09	1.01–1.18	0.025

* center 1: IRCCS Humanitas Research Hospital, Rozzano, Milan, Italy; center 2: Fondazione Policlinico Universitario A. Gemelli-IRCCS, Roma, Italy; center 3: Azienda Ospedaliero-Universitaria Pisana, Pisa, Italy; center 4: Tor Vergata University Hospital, Roma, Italy.
